# Differently Processed Low Doses of β-Glucan from Oat Bran Similarly Attenuate Postprandial Glycemic Response

**DOI:** 10.3390/foods13223623

**Published:** 2024-11-13

**Authors:** Denise Tan, Yueying Yao, Yifan Zhou, Chin Meng Khoo, Ludovic Penseyres, Andreas Rytz, Leroy Sivappiragasam Pakkiri, Chester Lee Drum, Jung Eun Kim, Kim-Anne Lê

**Affiliations:** 1 Department of Food Science and Technology, Faculty of Science, National University of Singapore, Singapore 117543, Singapore; denise_tan@u.nus.edu (D.T.); e0729489@u.nus.edu (Y.Y.); e0729752@u.nus.edu (Y.Z.); 2 Science and Technology Department, Nestlé R&D Center (Pte) Ltd., Singapore 618802, Singapore; 3 Division of Endocrinology, National University Hospital, University Medicine Cluster, Singapore 119074, Singapore; mdckcm@nus.edu.sg; 4 Department of Nutrition Sciences, Nestlé Research, 1000 Lausanne, Switzerland; ludovic.penseyres@rd.nestle.com (L.P.); andreas.rytz@rdls.nestle.com (A.R.); 5 Cardiovascular Research Institute (CVRI), National University Health System (NUHS), 14 Medical Drive, MD6 Level 8, Singapore 117599, Singapore; mdclspk@nus.edu.sg (L.S.P.); mdccld@nus.edu.sg (C.L.D.); 6 Department of Medicine, Yong Loo Lin School of Medicine, National University of Singapore (NUS), Singapore 119228, Singapore; 7 Department of Biochemistry, National University of Singapore (NUS), 8 Medical Drive, MD7, Singapore 117596, Singapore

**Keywords:** low dose, oat β-glucan, molecular weight, extractability, enzymatic hydrolysis, extrusion, beverage, postprandial glycemic response, randomized controlled trails

## Abstract

Incorporating β-glucan-rich oat bran (OB) can attenuate postprandial glycemic response (PPGR) in solid foods, but its effect in liquid matrices is unclear. This study investigated the ability of differently processed low-dose-β-glucan-containing beverages to lower PPGR, and the mechanisms of action. Twenty participants consumed five malt beverages made from cocoa powder: intact OB (Intact), OB treated with enzymatic hydrolysis (EnzymA, EnzymB) or extrusion (Extr), or no OB (Ctrl). Four-hour postprandial incremental areas under the curve (iAUC) and peak incremental concentrations (iCmax) of glucose, insulin, glucagon-like peptide 1 (GLP-1), gastric inhibitory polypeptide (GIP), and paracetamol were evaluated. The molecular weight (MW) and extractability of the β-glucan in all the test products were also assessed. The three-hour glucose iAUC significantly decreased by −26%, −28%, −32%, and −38% in Intact, EnzymA, EnzymB, and Extr, respectively, and the insulin levels of the oat-containing products were also significantly lower compared to Ctrl. Intact and Extr elicited a lower insulin iCmax and GLP-1 3 h iAUC compared to Ctrl. However, the GIP and paracetamol levels were not changed. All the processed OBs improved β-glucan extractability and lowered the MW of β-glucan compared to Intact. In conclusion, low-dose oat β-glucan in a beverage significantly reduced PPGR, with effects maintained across different oat processing methods.

## 1. Introduction

The growing prevalence of cardiometabolic diseases such as type 2 diabetes mellitus (T2DM) worldwide is well-documented [1,2]. High postprandial glycemic response (PPGR) and postprandial insulin response (PPIR) are considered major risk factors for the development of T2DM [3] and, in particular, foods high in readily digestible carbohydrates and low in dietary fiber tend to elicit a rapid and pronounced PPGR, accompanied by an increased release of insulin [4].

Carbohydrate content and quality, both at the food product level and within the overall diet, are the primary predictors of PPGR. Higher carbohydrate quality can be achieved by consuming more whole-food sources rich in natural dietary fibers, and/or by decreasing simple sugars [5]. This has led to the proposed concept of the “carbohydrates quality ratio”, which specifies that a product should contain at least 1 g of fiber and not more than 2 g of free sugars per 10 g of carbohydrates to contribute positively to dietary quality and reduce risks associated with poor glycemic control [6]. Amongst different fibers, cereal fibers have been shown to have stronger positive effects [7]. Favoring cereal sources of fibers in food products would therefore be of prime interest to lower PPGR, and ultimately contribute to lowering the risk for T2DM.

Amongst cereal sources, the consumption of oat bran has been identified as reducing PPGR owing to its high β-glucan content [8]. β-glucans are flexible, linear, unbranched β-(1-3), (1-4)-D-glucan polysaccharides, and this allows them to form hydrogen bonds amongst their own chains and with water molecules, conferring a high water-binding capacity. As a result, their consumption increases gastrointestinal viscosity, slowing gastrointestinal transit time, reducing the mixing of intestinal luminal contents, and hindering enzymatic digestion. This further delays the digestion of available carbohydrates (ACHO) and the transport of glucose to the intestinal epithelium for absorption [9]. Therefore, the European Food Safety Authority and other health authorities have confirmed the clinical evidence to be sufficiently strong, with several health claims approved for when β-glucans are applied at the specified dose of 4 g per 30 g of ACHO content [10,11].

While β-glucan can be more easily incorporated into solid products with relative effectiveness, depending on the doses, this is much more challenging in beverages due to β-glucan’s negative implications for beverage texture [12]. In addition, the beneficial effect of β-glucan has been proven to be more limited, and strongly dependent on β-glucan’s molecular weight and product viscosity [13]. In addition, while the mechanism of how β-glucans attenuate PPGR in solid foods is well-established, it is unknown whether a similar effect will be established at a low dose, particularly in a liquid matrix, where β-glucan has a diminished effect on gastrointestinal transit time. Oat bran processed through enzymatic hydrolysis has demonstrated the ability to overcome these sensory challenges and shows promise in improving beverages’ nutritional quality and predicted in silico glycemic properties, referring to a lower glucose release after in vitro digestion, at a β-glucan dose of 1.7 g per 30 g of ACHO [14]. However, previous studies have shown that the hydrolysis of β-glucans to make them more easy to incorporate into liquid matrices can result in the high release of maltose, maltotriose, and malto-oligosaccharides, as products of hydrolysis, thus negating all the potential beneficial effects [15]. Therefore, the present study aimed to clinically test the ability of β-glucan processed through different technologies to lower PPGR compared to a high-available-carbohydrates control beverage and, further, to investigate the mechanisms of action, by measuring the full carbohydrate profiles, as well as the physical–chemical properties of the different oat brans.

## 2. Materials and Methods

### 2.1. Test Products

The materials and methods used for the production of the 5 test products are described in the companion paper [5]. In brief, the materials included malt extract (Nestlé, Singapore), cocoa powder (JB Cocoa, Gelang Patah, Malaysia), skimmed milk powder (Vreugdenhil Groep BV, Maasdijk, The Netherlands), sucrose (Kong Hwa Trading, Singapore), palm olein (Ngo Chew Hong Edible Oil, Singapore), high-β-glucan oat bran OatWell^®^ 22 XF (DSM, Kaiseraugst, Switzerland), maltodextrin (dextrose equivalent 8.5) (Roquette, Lestrem, France), and water. Five cocoa malt beverage products were used, each containing a variable ingredient at a 12% dose:

i. Oat bran enzymatically hydrolyzed with protease and α-amylase (EnzymA);
ii. Oat bran enzymatically hydrolyzed with a protease, α-amylase, and amyloglucosidase (EnzymB);
iii. Oat bran processed by extrusion cooking (Extr);
iv. Oat bran with no further processing (positive control, Intact);
v. Maltodextrin (Ctrl) (negative control sample with no oat bran).

The enzymes used for the processing of oat bran were a protease (Novonesis, Kalundborg, Denmark), α-amylase (DSM, The Netherlands), and amyloglucosidase (Novonesis, Denmark). The detailed nutritional composition of our test products is described in Table 1.

### 2.2. Assessment of β-Glucan Properties and In Vitro Digestion Viscosity

The assessment of the β-glucan molecular weight (MW) was carried out by high-performance size-exclusion chromatography based on Ajithkumar et al. [6]. A standard oat flour control (Megazyme International, Bray, Ireland) was used for verification of results. β-glucan extractability was assessed through simulated digestion of the sample using a validated in vitro gastrointestinal model described by Brodkorb et al. [7]. Upon completion of intestinal incubation phase, in vitro intestinal digesta were centrifuged at 2000 g for 15 min at 37 °C. The β-glucan content of the supernatant was measured using AOAC 995.16 [8] and calculated with the below formula:

β-glucan extracted (%) = β-glucan in supernatant extract (g) ÷ β-glucan used in in vitro digestion (g) × 100.

where β-glucan used in in vitro digestion can be obtained from sample β-glucan content initially analyzed in [5].

In vitro digestion viscosity of digesta was also assessed using the in vitro digestion model mentioned above. In brief, gastric and intestinal digesta were extracted upon incubation at the respective phases and viscosity was assessed at 37 °C over shear rate range for digestion [9].

### 2.3. Randomized Human Trial

#### 2.3.1. Participants

The present study was registered with clinicatrials.gov as NCT04930250 and was approved by National Healthcare Group Domain Specific Review Board (2020/01464; date of approval: 30 April 2021) and National University of Singapore Institutional Review Board (NUS-IRB-2020-796; date of approval: 22 June 2021).

**Figure 1:**
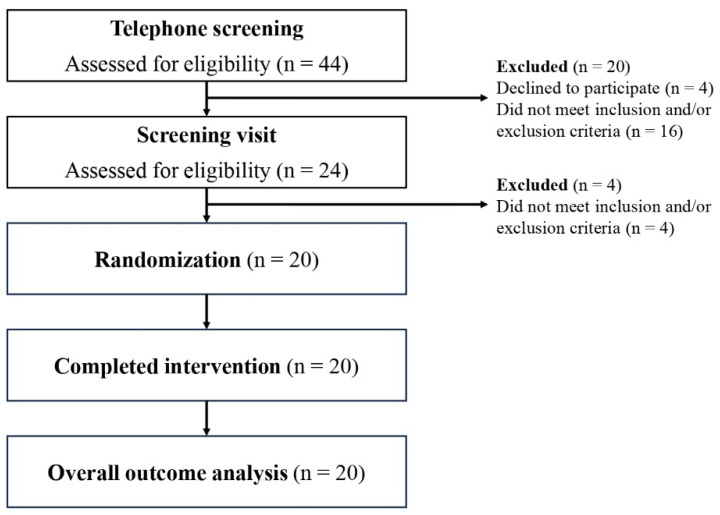
Participant flow diagram.

Participants were recruited via physical posters, electronic advertisements, and word of mouth between July 2021 and November 2021, from Singapore. To assess their eligibility and interest in the study, individuals who responded to the study advertisement were contacted for a telephone interview (*n* = 44). Eligible participants were asked to attend an in-person screening visit (*n* = 24) at the Investigational Medicine Unit (IMU), National University Hospital. During the screening visit, after providing informed consent, participants were assessed for their eligibility for the study through a structured questionnaire and basic health screening, including anthropometric and blood pressure measurements. Twenty healthy male and female participants, aged 24 to 39 years, were enrolled in this study, as they meet the following inclusion criteria: (1) body mass index (BMI) between 18.5 and 25 kg/m^2^; (2) able to understand and willing to sign an informed consent form in English; (3) regularly consume breakfast; (4) able and willing to consume 330 mL of liquid in 10 min; (5) for female participants, have a regular menstrual cycle and not be pregnant and lactating; (6) non-smokers; (7) no known food allergies or intolerances specifically to gluten, milk, lactose, or any grains; (8) no known drug allergies, specifically paracetamol. The participant recruitment process is summarized in Figure 1.

#### 2.3.2. Study Design

The present study was a randomized, double-blind, controlled crossover study. One researcher (D.T.) enrolled all the participants and all researchers involved in this study were blinded. All participants received 5 test products over 5 test visits, with 4–7-day washout period in between. Participants consumed 1 of the 5 test products at each study visit, with the products group-coded by an unblinded member of the study team, and the order of consumption randomized in a Latin square Williams design [10]. Participants were required to finish consuming the test product within 10 min. All female participants commenced their first test visit in the luteal phase of their menstrual cycle. The full study lasted 24–48 days for each participant.

#### 2.3.3. Procedure

Participants were instructed to avoid consuming high-fiber foods (i.e., whole grains, fruits, and vegetables) on the day prior to each study visit. At each study visit, participants reported to the IMU after a 10–12 h overnight fast. A concomitant diet and treatment review was performed, and vital sign (blood pressure) and anthropometric measurements (BMI, waist circumference) were taken to assess subjects’ compliance and safety. Each subject was provided with 50 g of oat-containing product reconstituted in 330 mL of water after fasting-state blood drawn. In total, 1000 mg of paracetamol in powder form were thoroughly mixed with the test products before being provided to each participant for consumption.

**Figure 2:**
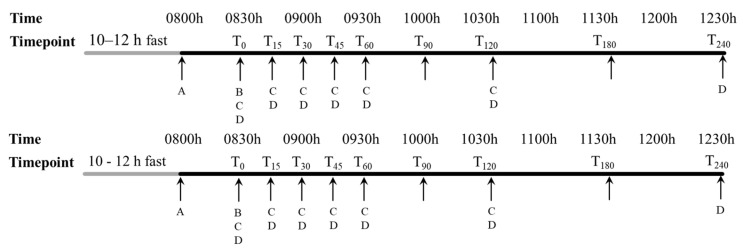
Study schedule for each test visit. Participants arrived after a 10–12 h overnight fast. A concomitant diet and treatment review was performed, and blood pressure and anthropometric measurements were taken to assess subject compliance and safety (A). Participants were cannulated and administered with the test product with paracetamol (B). Blood samples were taken at the respective timepoints for the measurement of blood glucose, insulin, active gastric inhibitory polypeptide, and active glucagon-like peptide-1 concentration (C). Blood samples were taken at the respective timepoints for the measurement of blood paracetamol concentration (D).

Blood samples were collected by a phlebotomist with participants in fasting state (T_0_) and at timepoints specified in Figure 2 (including T_15_, T_30_, T_45_, T_60_, T_90_, T_120_, T_180_, and T_240_) over 4 h post-ingestion to test blood glucose, insulin, active gastric inhibitory polypeptide, and active glucagon-like peptide-1 concentration. Blood was collected by intravenous cannulation via the antecubital vein into 4 types of blood collection tubes (Becton Dickinson): (i) sodium-fluoride-containing tubes, (ii) serum separator tubes, (iii) K2EDTA-containing tubes, and (iv) P800 tubes containing K2EDTA and proprietary additives. Blood samples for serum collection were left upright for 30 min before centrifugation, whilst blood samples for plasma collection were centrifuged within 15 min of collection. All samples were centrifuged at 3000× *g* for 15 min at 4 °C. Plasma and serum samples that were not used for on-the-day analysis were stored at −80 °C until sample analysis.

#### 2.3.4. Outcomes

The primary outcome of this study was to determine differences in peak incremental concentration (iC_max_) and incremental areas under the curve (iAUC) of blood glucose concentration between test products. The latter were calculated based on Wolever et al. [11], disregarding the negative area under baseline. To understand the physiological changes responsible for any differences in PPGR, hormones relevant to carbohydrate digestion and metabolism—insulin, active forms of glucagon-like peptide 1 (GLP-1), and active forms of gastric inhibitory polypeptide (GIP)—were also evaluated. Additionally, plasma paracetamol concentration was used as a marker of gastric emptying rate [12].

Plasma glucose concentration was quantified on the day of blood collection by hexokinase method (Roche Cobas) [13] and serum insulin concentration was analyzed using electrochemiluminescence immunoassay [14] by INNOQUEST Laboratories. Plasma concentrations of the active forms of GLP-1 and GIP were quantified by enzyme-linked immunosorbent assay (Immuno-Biological Laboratories Co., Ltd., Japan) [15]. Plasma paracetamol concentration was quantified using liquid chromatography–tandem mass spectrometry [16].

#### 2.3.5. Power Calculation and Statistical Analysis

Based on a prior pilot study of 16 participants (12 males, 4 females) who were metabolically healthy (unpublished), a 29% reduction in glucose response 2 h iAUC (*p* = 0.04) was found after oat bran was added to a cocoa malted beverage of a similar recipe versus a reference in which sucrose was replaced. With the assumption of a similar response, the sample size was calculated using the following formula:

$$n=(\frac{Z_{1-\alpha /2}+Z_{\beta }}{\Delta /\sigma })$$

where *α* = 0.05 (two-tailed), *β* = 0.20 for 80% power, Δ = 29% estimated effect size, and *σ* is the estimated standard deviation of the response. This calculation indicated that ≥16 participants would be required. Taking into account a 20% dropout rate, 20 participants were recruited for the present study.

Data analysis was processed with a blinded researcher, with unblinding occurring only after the statistical analysis was completed. Glucose, insulin, GIP, GLP-1, and paracetamol were analyzed as iAUC, since these outcomes were expected to increase following test product consumption. Differences in β-glucan characteristics (extractability and MW) and in vitro gastric and intestinal digesta viscosity between test products were assessed by one-way analysis of variance (ANOVA), since only one variable (intervention) was presented in five test products. Differences in physiological markers, including glucose, insulin, GIP, GLP-1, and paracetamol, were assessed using a one-way repeated-measures ANOVA [17] to determine differences between interventions at each time point. Tukey’s post hoc test was carried out to ascertain differences between test products and collected blood samples. A Pearson correlation was used to assess the association between glucose response 3 h iAUC and β-glucan MW. All results are presented as mean ± standard error of mean (SEM) and a *p*-value < 0.05 was used to determine statistical significance. All data and figures were processed in Microsoft Excel (2019) and all statistical analyses were carried out with STATA 13 (StataCorp LLC, College Station, TX, USA).

## 3. Results

### 3.1. β-Glucan Properties and In Vitro Digestion Viscosity

The β-glucan extractability results are reported in Table 1. Compared to the unprocessed oat bran, Intact, all the products containing the additionally processed oat bran (EnzymA, EnzymB, and Extr) had an improved β-glucan extractability (31% vs. 38–50%) (*p* = 0.000). The results of the β-glucan MW can be found in Supplementary Figure S1 and Table 1, and relative to the Intact, there were 41%, 57%, and 16% loss in peak β-glucan MW in the EnzymA, EnzymB, and Extr, respectively (*p* = 0.000). Table 2 depicts the in vitro gastric and intestinal viscosity results at 60 s^−1^, 37 °C. Extr had the highest in vitro gastric viscosity, followed by Intact, EnzymA, Ctrl, and EnzymB (*p* = 0.000). No difference in in vitro intestinal viscosity was observed.

### 3.2. Randomized Human Study

#### 3.2.1. Participant Characteristics

The characteristics of the study participants are detailed in Table 3. All 20 participants (10 men and 10 women; mean age: 29 y) were healthy, with their BMI, waist circumference, blood pressure, fasting glucose, and fasting insulin concentrations within a healthy range [1,2,18,19]. The majority (90%) of the participants were ethnically Chinese.

#### 3.2.2. Physiological Outcomes

**Figure 3:**
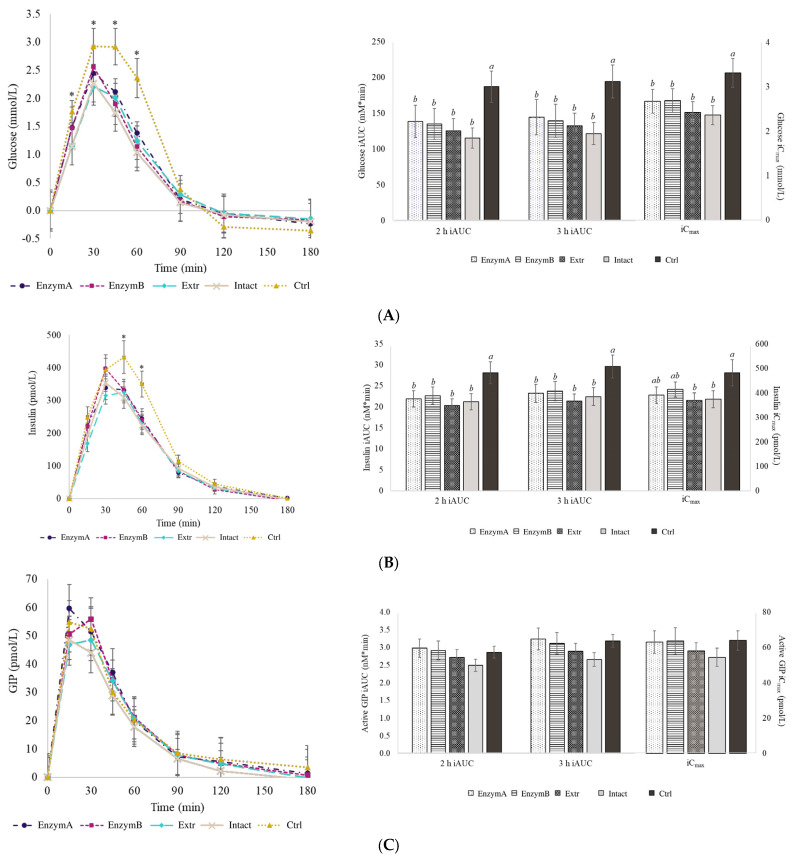

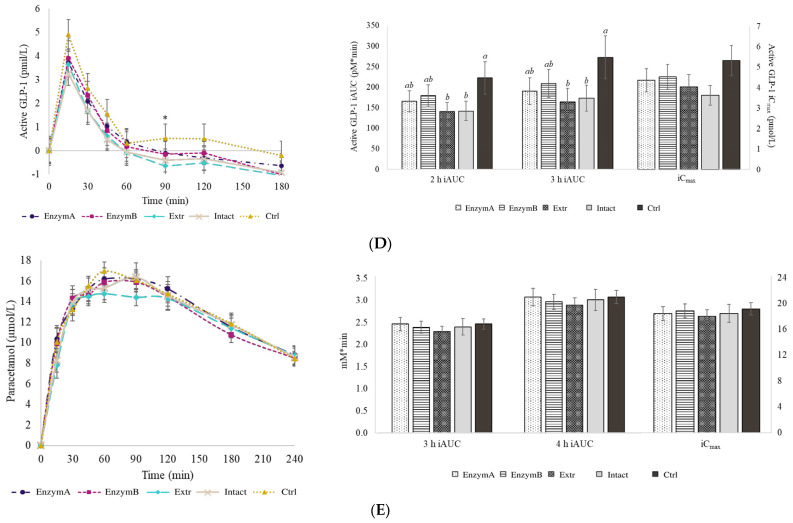
Line graphs depict the change in blood (**A**) glucose, (**B**) insulin, (**C**) GIP, (**D**) GLP-1, and (**E**) paracetamol concentrations following administration of each test product. Values with asterisk (*) indicate a significant difference between at least two treatments (at *p* < 0.05; one-way repeated-measures ANOVA with Tukey’s post hoc). Bar charts show the iAUC and iCmax for each biomarker. Within each bar graph, bars without a common letter denote a significant difference (at *p* < 0.05; one-way repeated-measures ANOVA with Tukey’s post hoc), where a indicates the highest value across the property. Values are mean ± SEM (*n* = 20). Abbreviations: iAUC, incremental area under the curve; iCmax (peak incremental response); GIP, glucose-dependent insulinotropic polypeptide; GLP-1, glucagon-like peptide-1; EnzymA, oat bran enzymatically hydrolyzed with a protease and α-amylase; EnzymB, oat bran enzymatically hydrolyzed with a protease, α-amylase, and amyloglucosidase; Extr, oat bran processed by extrusion cooking; Intact, oat bran with no further processing (positive control); Ctrl, maltodextrin (negative control sample with no oat bran).

The effects of the five oat-containing products on the blood glucose, insulin, active GIP, active GLP-1, and paracetamol concentrations are shown in Figure 3A–E. No significant differences were observed for the baseline values between the different study days.

The three-hour iAUC-glucose values of the oat-containing products (−26% for EnzymA, −28% for EnzymB, −32% for Extr, and −38% for Intact, respectively) were significantly lower compared to that of Ctrl (*p* < 0.001). Differences between the oat-containing products and Ctrl were also observed at T = 45 min (*p* < 0.001) and T = 60 min (*p* < 0.001), and with the iC_max_ (*p* < 0.001) and 2 h iAUC (*p* < 0.001). This was accompanied by the similar trend in serum insulin: T = 45 min (*p* = 0.003), T = 60 min (*p* = 0.000), 2 h iAUC (*p* = 0.000), and 3 h iAUC (*p* = 0.000). However, whilst Intact (*p* = 0.022) and Extr (*p* = 0.015) had lower insulin iC_max_ compared to Ctrl, this difference remained non-significant for EnzymA (*p* = 0.075) and TP-2 (*p* = 0.293).

Significantly lower active GLP-1 2 h iAUC (*p* = 0.028) and 3 h iAUC (*p* = 0.022) were observed with Intact and Extr compared to Ctrl, although this was not significant for EnzymA (*p* = 0.261) or TP-2 (*p* = 0.528). No differences in the kinetic parameters (iAUC and iCmax) of the active GIP or paracetamol concentrations were found across the products.

#### 3.2.3. Correlation Between β-Glucan Molecular Weight and PPGR

A significant negative correlation was identified between 3 h iAUC-glucose and β-glucan MW (r = −0.93; *p* = 0.020), and this result is shown in Supplementary Figure S2.

## 4. Discussion

In this study, we investigated the effects of a beverage containing a low dose of β-glucan on PPGR and associated biomarkers, and various processing methods were also applied to the oat bran to assess differential responses on PPGR, accordingly. Our findings suggested that all the processing methods could improve β-glucan extractability and lower the MW of β-glucan, which was associated with similar PPGR-lowering effects amongst the different processing methods, despite the distinct nutritional profiles and physicochemical properties of the different oat brans. Moreover, the enhancement of carbohydrate quality through the incorporation of low-dose oat β-glucan as a bulking agent in beverages can yield significant health benefits by lowering PPGR. This improvement in glycemic control is vital for disease prevention, as it may mitigate the risks associated with poor glycemic regulation and contribute to a reduced incidence of metabolic disorders, including type 2 diabetes [20].

It was previously suggested that the specific physicochemical properties of β-glucan are responsible for its effectiveness in lowering glycemic response, which most studies attribute to the viscosity-conferring effect of the polysaccharide [21,22]. However, the results from this study show that this may not be the key mechanism through which β-glucan attenuates PPGR in liquid meals. Intact and Extr were observed to have 9- and 11-times greater in vitro gastric viscosity compared to Ctrl, respectively, although this did not lead to a significantly slower gastric emptying rate or lower PPGR in vivo. Potentially, liquid meals may only have a clinically relevant impact on gastric viscosity during the immediate period post-consumption [23], which may explain why liquid products, despite having a relatively high viscosity, still have no direct correlation with gastric emptying rates or effects on PPGR attenuation. Other studies have also indicated that the increased viscosity of liquid β-glucan-containing foods does not determine its effect on glycemic control [24,25], which contrasts with observations made in semi-solid [26] or solid [27] β-glucan-containing foods.

Instead, it has been observed that β-glucan MW, rather than beverage viscosity, determines glycemic response [25], and this previous finding aligns with our observation of an inverse correlation between the glucose response of 3 h iAUC and β-glucan MW. This is possibly because gastric viscosity and emptying rate are regulated by a variety of factors, including meal composition and matrix [28]. Generally, a highβ-glucan MW can slow down the diffusion of glucose in the small intestine by trapping digestive enzymes, including amylase, within its network, thereby weakening the glycemic response [29]. Therefore, a loss of β-glucan MW could impair this property, as the network would not form as effectively [25,29]. In this study, there was a minor loss of 15.9% β-glucan MW post-extrusion in Extr, and a meaningful loss after enzymatic hydrolysis in EnzymA and TP-2. However, we still observed PPGR attenuation with meaningful MW reduction after processing, and this may be attributed to the ability of β-glucan chains that have been broken due to hydrolysis to re-form connections within the network upon re-solubilization in the beverage [30,31]. Furthermore, a MW of 580 kDa has been found to attenuate PPGR in a beverage matrix [24]. This provides confidence in the idea that the current oat processing methods do not damage β-glucan extensively, as the lowest achieved peak MW of the processed oat brans is 819 kDa. Moreover, all the processing methods increased the β-glucan extractability of the oat bran, increasing the concentration of available β-glucan interacting with the intestinal digesta. This interaction could form a viscous gel that may physically hinder the digestion and absorption of carbohydrates, ultimately slowing glucose release into the bloodstream and attenuating the PPGR [32]. GLP-1, which is an incretin hormone, is secreted by intestinal endocrine L-cells in response to the presence of nutrients, with greater sensitivity to carbohydrates and lipids, and could slow gastric emptying to control postprandial blood glucose concentrations [33]. In this study, a lower GLP-1 concentration was observed upon consumption of Intact and Extr compared to Ctrl, likely due to the lower carbohydrate content and higher early phase gastric viscosity of Intact and Extr. This was not observed between EnzymA and EnzymB versus EnzymA, potentially due to their similarities in gastric viscosity. As mentioned earlier, the viscosity of liquid meals and their gastric emptying rate do not affect PPGR as much as β-glucan MW, which explains why, despite the reduction in GLP-1 in Intact and Extr, the attenuation of PPGR still persisted. Additionally, there were no differences observed in GIP concentration among our test products. This suggests that the PPGR-attenuation effect of our test products may be independent of GIP, aligning with the findings from other studies [34].

Although dietary protein has been shown to improve insulin sensitivity by regulating hormones and glycemic response, this study observed a similar trend in insulin response to glycemic response, despite the oat-containing beverages containing a slightly higher level of dietary protein. This lack of difference may be attributed to the fact that the additional protein content was negligible, and therefore did not elicit a clinically relevant difference. In our test products, the maximum difference in protein content was no more than 3.1%, and as demonstrated in a previous study, a 15% difference in protein content between two meals failed to have a significant effect on postprandial plasma blood glucose or insulin responses [35].

This study has several strengths. A randomized controlled trial design with relevant secondary endpoints alongside a concurrent evaluation of β-glucan physiochemical properties provided an insight into the mechanism of action through which processed oat attenuates PPGR in liquid meals. Additionally, studying the PPGR-attenuating effect of low-dose oat β-glucan contributes to the development of healthy and palatable beverages. Last, but not least, unlike many studies assessing the impact of β-glucan in an aqueous solution, the test product employed was a beverage formulated to simulate real-life conditions in which oats and β-glucan may interact with other nutrients and influence physiological outcomes. However, we also noticed several limitations in this study. It was not controlled for ACHO content across test products and, thus, could not verify the contribution of glucose-responsive iAUC reduction due to ACHO differences versus β-glucan in each sample. However, to provide a realistic representation of product development, this study controlled for weight across products instead. The differences in ACHO across the test products should be carefully considered when interpreting the results. Further studies controlling for ACHO content can help to verify the contribution for each processed oat bran. Additionally, this study employed postprandial paracetamol blood response to evaluate gastric emptying, which might be affected by factors other than the actual emptying rate. Hence, it is possible that a difference in gastric emptying may have been found for Ctrl versus Intact and Extr in the first 15 min of consumption if the gold standard of gastric emptying, scintigraphy [36], had been used, the population size had been bigger, or the marker had also been assessed at earlier timepoints (e.g., 5 min and 10 min post-consumption). Further studies are warranted to assess the osmolarity of the beverage products to aid in the interpretation of their gastric emptying rates and to enhance their downstream clinical applications [37]. Another potential limitation may be the recruited participants, who were metabolically healthy and lean adults. This limits the generalizability of the present results to a broader population demographics.

## 5. Conclusions

This study demonstrated the potential to improve the carbohydrate quality of a beverage by increasing its cereal fiber content, with positive effects on lowering PPGR, while maintaining low product viscosity. Mechanisms other than β-glucan molecular weight (MW), gastric viscosity, and delayed gastric emptying may be responsible for the lower PPGR. These findings may create an opportunity for the use of oat fibers as healthy and nutritious bulking agents to lower PPGR in beverages.

## Figures and Tables

**Table 1:** Nutrient information of cocoa malt beverage powder products.

Nutrient	g/100 g				
	EnzymA	EnzymB	Extr	Intact	Ctrl
Total CHO	69.58 ± 0.13 ^b^	69.35 ± 0.06 ^b^	69.94 ± 0.01 ^b^	69.49 ± 0.33 ^b^	73.45 ± 0.15 ^a^
ACHO	57.68 ± 0.13 ^b^	57.50 ± 0.12 ^b^	58.29 ± 0.19 ^b^	57.89 ± 0.18 ^b^	67.32 ± 0.16 ^a^
Total dietary fiber	11.90 ± 0.00 ^a^	11.85 ± 0.18 ^a^	11.65 ± 0.18 ^a^	11.60 ± 0.14 ^a^	6.13 ± 0.01 ^b^
β-glucan	3.51 ± 0.01 ^ab^	3.66 ± 0.05 ^a^	3.34 ± 0.04 ^b^	3.40 ± 0.05 ^ab^	0.19 ± 0.01 ^c^
β-glucan extractability of sample, %	37.7 ± 1.6 ^b^	50.0 ± 0.5 ^a^	39.2 ± 2.2 ^ab^	31.4 ± 0.0 ^c^	N.A.
Peak β-glucan MW of flour, kDa	1135 ± 6 ^c^	819 ± 1 ^d^	1616 ± 8 ^b^	1921 ± 39 ^a^	N.A.
Protein	15.1 ± 0.00 ^a^	15.05 ± 0.11 ^a^	15.35 ± 0.04 ^a^	15.35 ± 0.25 ^a^	12.25 ± 0.04 ^b^
Fat	9.55 ± 0.11	9.80 ± 0.14	9.50 ± 0.00	9.45 ± 0.04	9.05 ± 0.11

Results presented as mean ± SEM. Different alphabet superscript indicates a significant difference (*p* < 0.05) across the same row. Superscript a indicates the highest-value group across the same row. Abbreviations: ACHO, available carbohydrates; CHO, carbohydrates; EnzymA, oat bran enzymatically hydrolyzed with a protease and α-amylase; EnzymB, oat bran enzymatically hydrolyzed with a protease, α-amylase, and amyloglucosidase; Extr, oat bran processed by extrusion cooking; Intact, oat bran with no further processing (positive control); Ctrl, maltodextrin (negative control sample with no oat bran); MW, molecular weight. *n* = 2 for EnzymA, EnzymB, Extr, Intact, Ctrl.

**Table 2:** In vitro gastric and intestinal viscosity at 37 °C and a shear rate of 60 s^−1^.

	In Vitro Gastric Viscosity (mPa.s)	In Vitro Intestinal Viscosity (mPa.s)
EnzymA	2.60 ± 0.07 ^c^	1.27 ± 0.08
EnzymB	1.56 ± 0.01 ^d^	1.17 ± 0.01
Extr	15.28 ± 0.06 ^a^	1.36 ± 0.01
Intact	12.18 ± 0.07 ^b^	1.58 ± 0.08
Ctrl	1.38 ± 0.01 ^d^	1.10 ± 0.22

Values are mean ± SEM (*n* = 2). Values with different letters in a row are significantly different (at *p* < 0.05) based on one-way ANOVA and Tukey’s post hoc. Superscript a indicates the highest-value group across the same row. Abbreviations: EnzymA, oat bran enzymatically hydrolyzed with a protease and α-amylase; EnzymB, oat bran enzymatically hydrolyzed with a protease, α-amylase, and amyloglucosidase; Extr, oat bran processed by extrusion cooking; Intact—oat bran with no further processing (positive control); Ctrl, maltodextrin (negative control sample with no oat bran).

**Table 3:** Characteristics of study population.

Characteristics	Mean (±SEM)/*n*
Gender (*n*)	
Male	10
Female	10
Race (*n*)	
Chinese	18
Malay	1
Caucasian	1
Age (years)	29 ± 0.9
Blood pressure (mmHg)	
Systolic blood pressure	110.7 ± 2.2
Diastolic blood pressure	69.5 ± 1.2
BMI (kg/m^2^)	22.1 ± 0.3
Waist circumference (cm)	72.1 ± 1.2
Fasting glucose (mmol/L)	4.9 ± 0.0
Fasting insulin (pmol/L)	36.4 ± 1.5

Values are mean ± SEM (*n* = 20). Abbreviations: BMI, body mass index.

## Data Availability

Data for this article, including Table 1 and Table 2, are available at ScienceDirect at https://doi.org/10.1016/j.lwt.2023.114729 (accessed on 10 November 2024). Data collected from human participants, described in Table 3 and Figure 3, are not available for confidentiality reasons.

## Supplementary Materials

The following supporting information can be downloaded at: https://www.mdpi.com/article/10.3390/foods13223623/s1, Figure S1: Graph depicting β-glucan molecular weight of each test product; Figure S2: Correlation between peak β-glucan molecular weight and postprandial glycemic response iAUC 3 h.
