# Supplementary material for: Differently Processed Low Doses of β-Glucan from Oat Bran Similarly Attenuate Postprandial Glycemic Response
Source: Foods. 2024 Nov 13;13(22):3623. doi: 10.3390/foods13223623 (PMC11594264; doi:10.3390/foods13223623)
Supplement: Supplementary file 1 [file foods-13-03623-s001.zip › foods-3281638-supplementary.pdf]

Supplementary materials

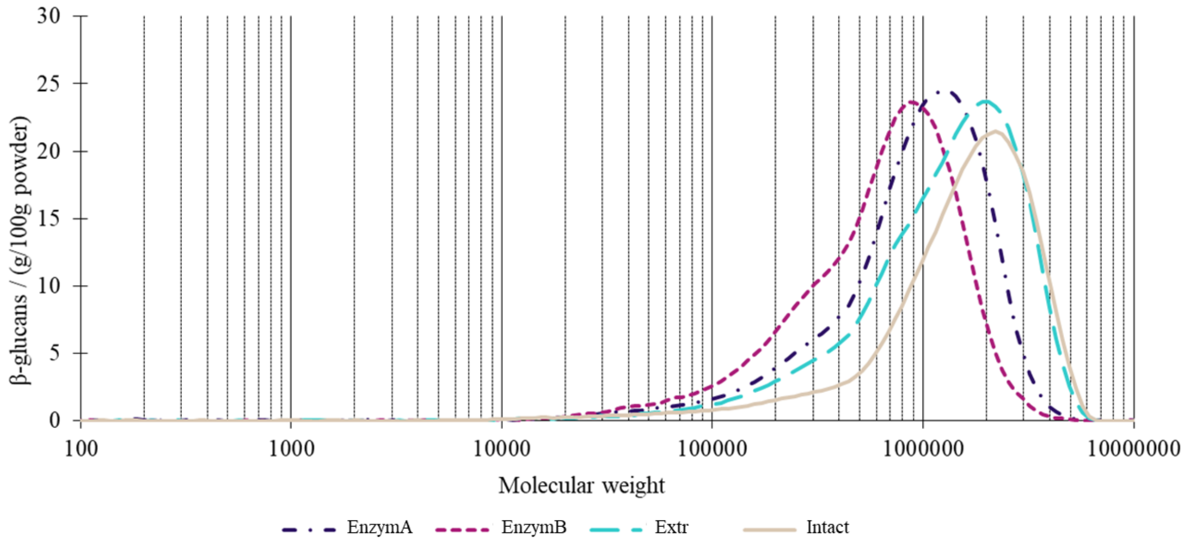

Figure S1: Graph depicting β-glucan molecular weight of each test product. Values are mean (n=2). Abbreviations: EnzymA, oat bran enzymatically hydrolysed with a protease and α-amylase; EnzymB, oat bran enzymatically hydrolysed with a protease, α-amylase and amyloglucosidase; Extr, oat bran processed by extrusion-cooking; Intact - oat bran with no further processing (positive control).

Supplementary materials

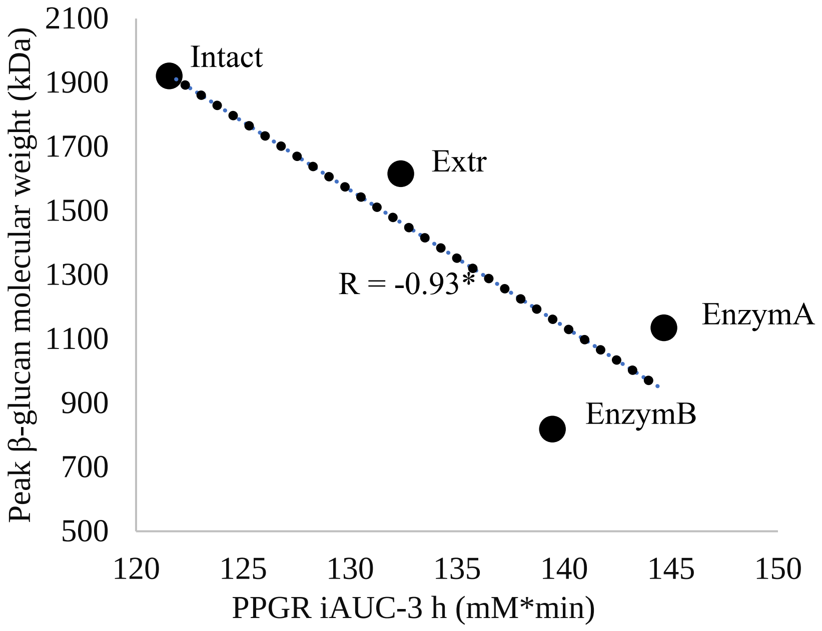

Figure S2: Correlation between peak β-glucan molecular weight and postprandial glycemic response iAUC-3h. Abbreviations: iAUC, incremental area under the curve; PPGR, postprandial glycemic response; EnzymA, oat bran enzymatically hydrolysed with a protease and α-amylase; EnzymB, oat bran enzymatically hydrolysed with a protease, α-amylase and amyloglucosidase; Extr, oat bran processed by extrusion-cooking; Intact - oat bran with no further processing (positive control).
